# Anal Canal Duplication With Abscess Formation and Associated Sacrococcygeal Hamartoma in an Adolescent: A Report of a Rare Case

**DOI:** 10.7759/cureus.85453

**Published:** 2025-06-06

**Authors:** Mohamad Al Ayoubi, Abbas Rachid, Abed AlRaouf Kawtharani, Sara Ayoub, Faten Hijazi

**Affiliations:** 1 Gastroenterology and Hepatology, Lebanese University, Beirut, LBN; 2 Internal Medicine, Lebanese University, Beirut, LBN; 3 Medical Sciences, Lebanese University, Beirut, LBN

**Keywords:** acessory anal canal, anal abscess, anal canal duplication, hamartoma, sacrococcygeal hamartoma

## Abstract

Anal canal duplication (ACD) is a rare congenital malformation, typically diagnosed in pediatric populations, and often associated with other anomalies. Due to its nonspecific symptoms, it may be misdiagnosed or diagnosed late, particularly in adolescents or adults.

We report the case of a 17-year-old female patient who presented with rectal pain, fever, and purulent perianal discharge. Physical examination revealed an accessory anal opening posterior to the native anus. Magnetic resonance imaging (MRI) demonstrated a perirectal abscess, a duplicated anal canal, and a suspicious presacral mass. A colonoscopy revealed a bulging rectum with normal mucosa. Surgical excision was performed for both the accessory anal canal and the presacral mass, and histopathological analysis confirmed the diagnosis of ACD and identified the mass as a benign sacrococcygeal hamartoma.

This case highlights the diagnostic challenge of ACD due to its clinical resemblance to other perianal pathologies. Imaging modalities, particularly MRI and endoscopy, are critical for excluding differential diagnoses, guiding surgical management, and confirming post-surgical histopathology. ACD itself is a rare condition, with abscess formation being a rarely reported complication. The co-occurrence of ACD and a sacrococcygeal hamartoma is exceptionally rare and, to our knowledge, has not been previously reported.

This case underscores the importance of a multidisciplinary approach for accurately diagnosing and effectively treating rare anorectal anomalies such as ACD, particularly when complicated by infection or associated with other anomalies.

## Introduction

Anal canal duplication (ACD) is a rare congenital malformation, with fewer than 100 cases reported in the literature, predominantly in pediatric populations. It is approximately nine times more prevalent in females than in males and is associated with other congenital abnormalities in up to 36% of cases. Due to its often subtle or asymptomatic presentation, ACD may remain undiagnosed until later in life. In some instances, symptoms can be mistaken for those of other anorectal conditions, such as perianal fistulas, dermoid cysts, presacral teratomas, lumbosacral meningoceles, or spina bifida [1,2]. Accessory ACD, first described by Dukes and Galvin in 1956 [3], is defined by the presence of an additional anal canal, typically situated along the midline and posterior to the normal anal canal.

ACD is a congenital malformation typically found in early infancy, characterized by a midline tubular structure located posterior to the anus. It does not communicate with the native anal canal and occurs more frequently in females [4].

In 2002, Ochiai et al. established the histopathological criteria for diagnosing ACD, which include (1) the presence of squamous epithelial cells at the caudal end, (2) transitional epithelium at the cranial end, and (3) smooth muscle cells within the lesion wall [2]. This condition is thought to result from the abnormal embryological development of the hindgut during the fifth to seventh weeks of gestation, involving duplication of the dorsal cloaca [4]. The clinical presentation of ACD is highly variable, ranging from asymptomatic accessory anal openings to more complex cases involving infections, abscesses, or perianal fistulas. Accurate diagnosis typically relies on advanced imaging modalities like magnetic resonance imaging (MRI) and endoscopic assessment, which help detect associated anomalies such as presacral masses or fistulous tracts [5]. In this case report, we describe a 17-year-old female patient diagnosed with ACD complicated by abscess formation. This case illustrates the diagnostic complexity of ACD and emphasizes the need for a multidisciplinary approach to ensure timely and effective management.

## Case presentation

A 17-year-old female patient presented with a five-day history of intermittent purulent discharge from the perianal region, accompanied by rectal pain and fever. She reported no identifiable aggravating or relieving factors. Despite completing a 72-hour course of oral metronidazole and cefixime, her symptoms persisted, prompting outpatient evaluation. On examination, her vital signs were stable except for a documented fever of 39.2°C.

**Figure 1:**
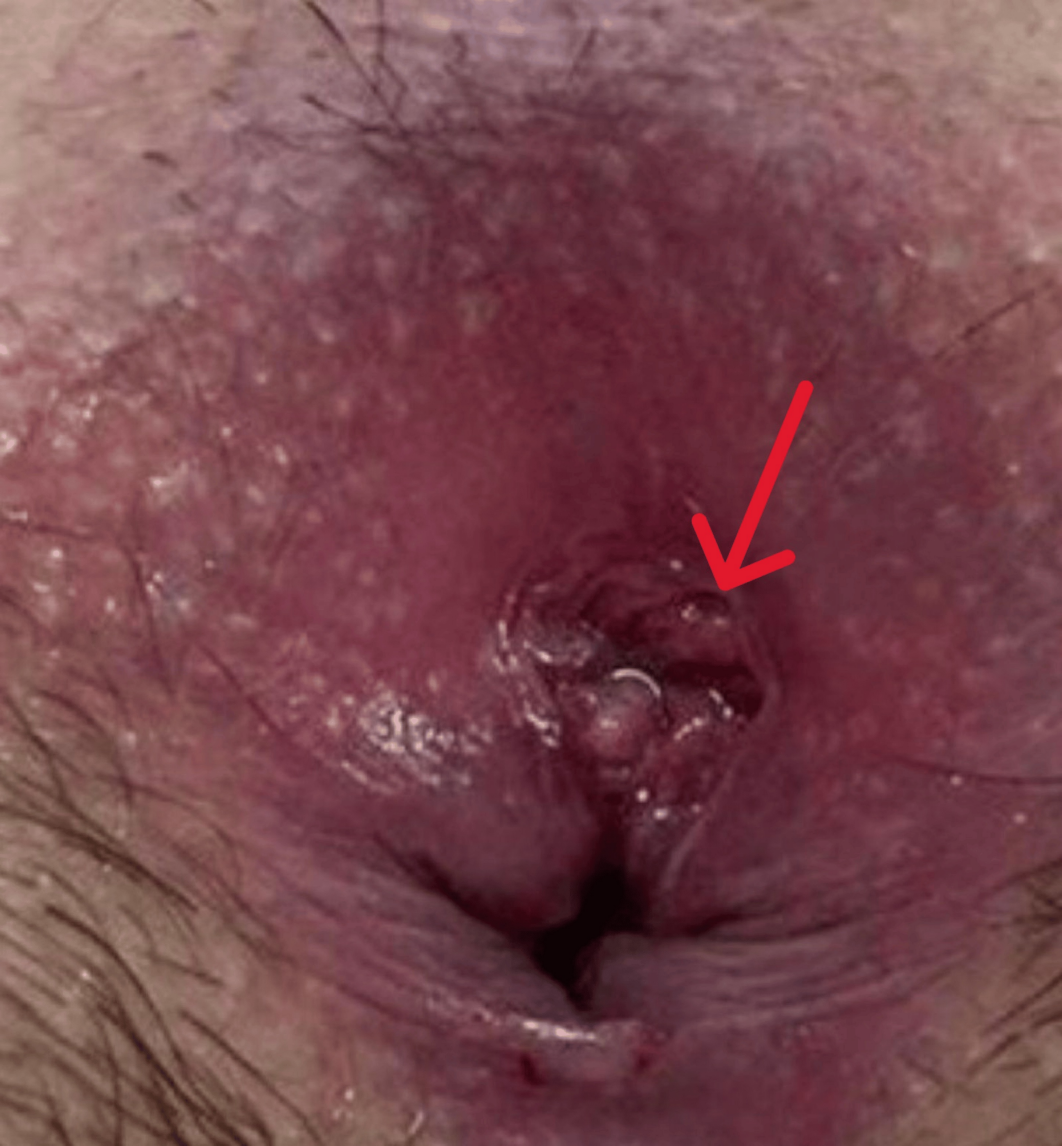
Image highlighting an accessory perianal orifice with dentate line-like features (arrow) suggestive of anal canal duplication

Physical examination revealed an accessory anal opening along the posterior midline, just above the native anal canal. The opening was associated with fecal debris and a malodorous discharge. The remainder of the systemic examination, including cardiovascular, respiratory, and neurological assessments, was unremarkable. Her medical and surgical history was noncontributory, and she denied any previous anorectal complaints, abscesses, or related issues. With consent, a photograph of the lesion (Figure 1), resembling a small secondary anus with dentate line-like features, was obtained for expert panel review.

Given the clinical findings, the gastroenterology team suspected ACD complicated by an abscess. The patient was admitted for further workup and management. Laboratory tests revealed signs of systemic inflammation, including elevated inflammatory markers (Table 1).

**Table 1:** Laboratory investigation of our patient during hospitalization WBC: white blood cells; CRP: C-reactive protein; SGOT: serum glutamic-oxaloacetic transaminase; GGT: gamma-glutamyl transferase

Parameter	Patient value	Reference range
WBC	3,800	4,500-11,000/mm^3^
Hemoglobin	10.6 g/dL	13-17.5 g/dL
Platelets	183,000	150-400 x 10^9^/L
CRP	60 mg/L	<5 mg/L
SGOT	14.9 U/L	5-31 U/L
GGT	8 U/L	6-42 U/L
Lipase	31.9 U/L	13-60 U/L

**Figure 2:**
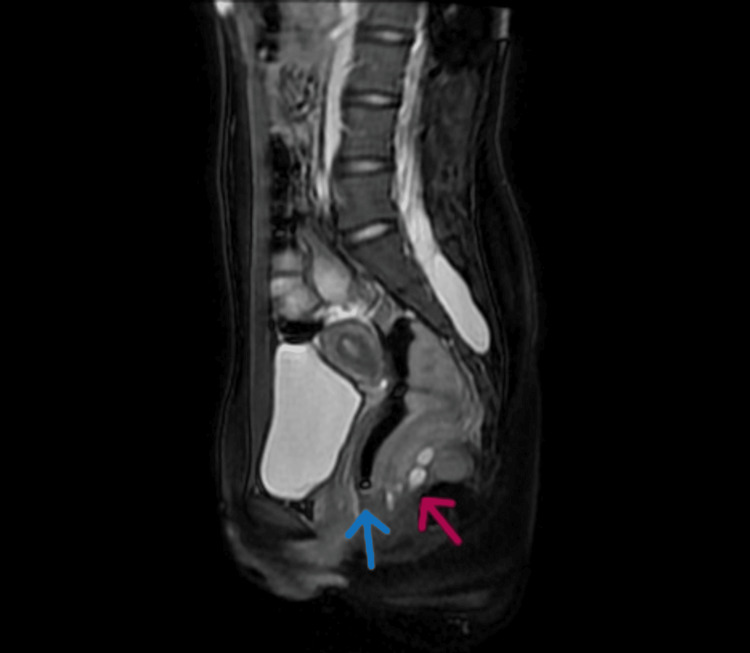
Sagittal 2D FIESTA MRI view demonstrating a well-defined perirectal collection with central necrosis, suggestive of perirectal abscess formation localized within an anal accessory canal (red arrow), without communication with the principal canal (blue arrow) 2D FIESTA MRI: two-dimensional fast imaging employing steady-state acquisition magnetic resonance imaging

MRI of the pelvis revealed a posterior tract to the anal canal without communication between them. It also demonstrated a well-defined collection measuring 4.5 × 3.5 × 3.5 cm with an enhancing wall and central necrosis located posterior to the rectum, suggestive of a perirectal abscess or a necrotic mass (Figure 2).

**Figure 3:**
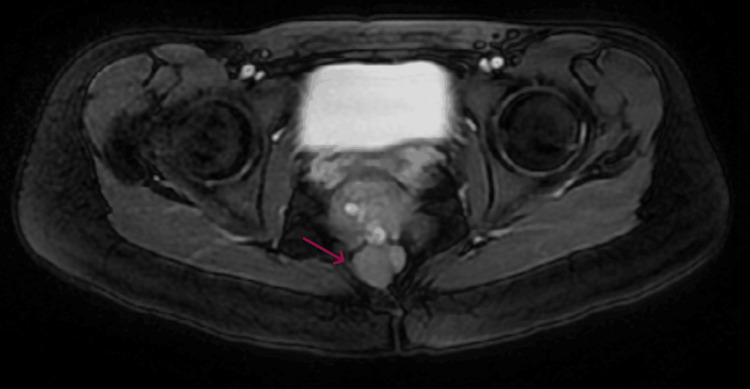
Axial 2D FIESTA MRI demonstrating a mildly enhancing solid lesion anterior to the coccyx, measuring 2.5 × 2 × 1.8 cm (red arrow) 2D FIESTA MRI: two-dimensional fast imaging employing steady-state acquisition magnetic resonance imaging

A separate, mildly enhancing solid lesion measuring 2.5 × 2 × 1.8 cm was also identified anterior to the coccyx, raising differential considerations such as a solid neoplasm or a presacral-precoccygeal phlegmon. Potential diagnoses, including perianal fistula, dermoid cyst, presacral teratoma, lumbosacral meningocele, and spina bifida, were considered but subsequently ruled out based on imaging findings (Figure 3).

The patient was started on intravenous antibiotics, including ceftriaxone and metronidazole, along with supportive care. Blood and urine cultures were obtained, with no microbial growth observed during the incubation period. Despite the absence of positive culture results, laboratory markers supported an active infectious or inflammatory process, consistent with the clinical and radiological findings. Following five days of intravenous antibiotic therapy, an anoscopy with hydrogen peroxide and methylene blue injection was performed to confirm the absence of communication with the anal canal. Subsequently, the patient underwent a colonoscopy under sedation with midazolam and fentanyl. The colonoscopy revealed a significantly bulging rectum with no internal fistulous orifice identified and otherwise normal mucosa. The remainder of the colon, including the sigmoid, descending, transverse, and ascending segments and the cecum, appeared normal with normal terminal ileum. These findings, combined with previous imaging and clinical presentation, confirmed the diagnosis of a secondary anal opening located along the posterior midline, consistent with ACD complicated by an abscess.

**Figure 4:**
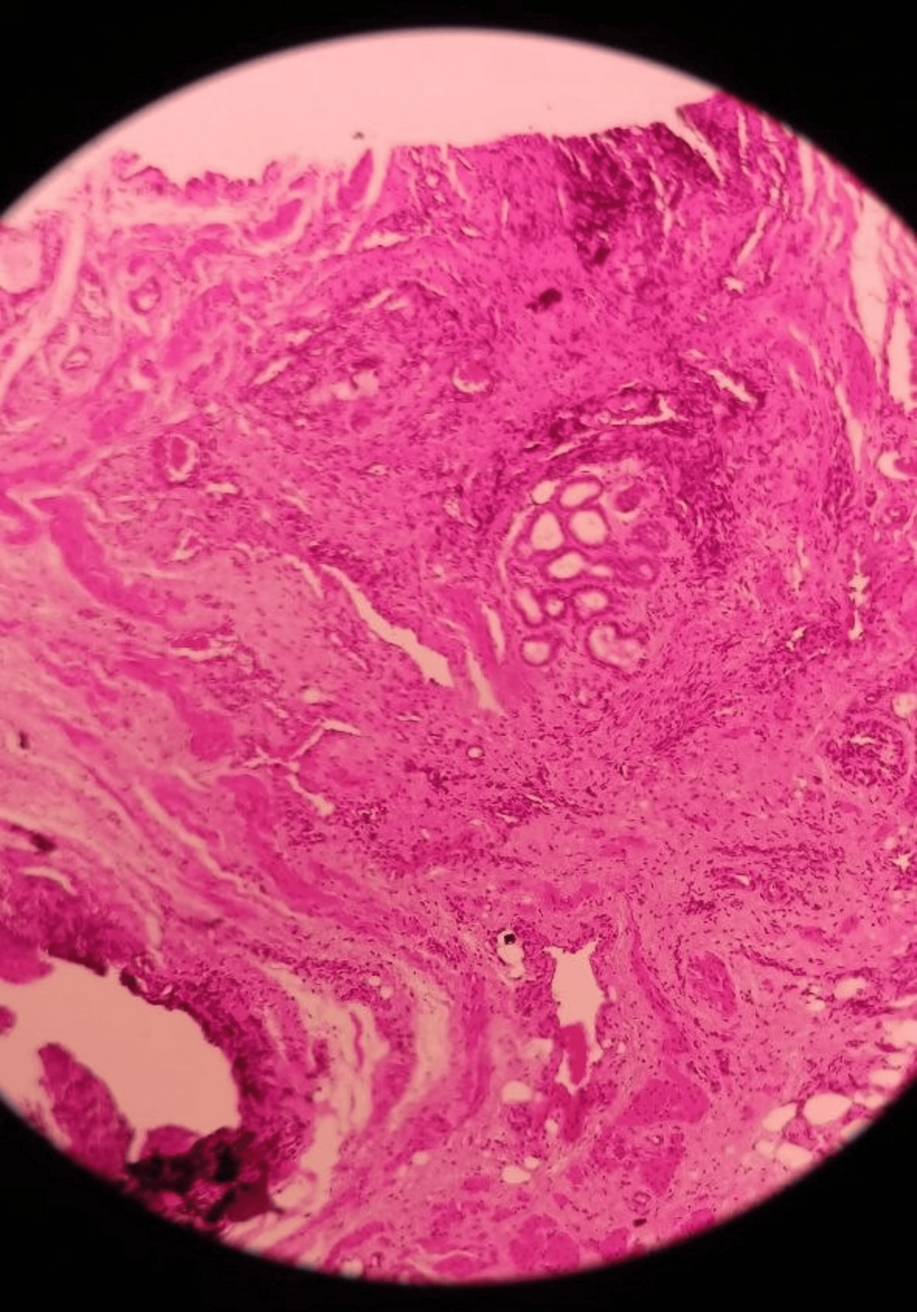
Histological composition of the hamartoma composed of squamous and columnar epithelium with fibrous stroma, skeletal muscle, and secretory gland aggregates

Perirectal abscess drainage was performed, followed by complete surgical excision of the duplicated anal canal and the associated presacral mass. Histopathological examination of the duplicated canal revealed normal anal transitional zone epithelium at the cranial end and squamous epithelium at the caudal end, consistent with the diagnostic criteria for ACD. Meanwhile, the biopsy of the mass located in the sacrococcygeal region demonstrated features of a benign hamartoma, composed of cysts lined by epithelial and squamous mucosa, fibrous and stromal tissue, and adipose tissue with prominent vasculature. These histological findings, in conjunction with the clinical and imaging data, confirmed the rare coexistence of ACD and a sacrococcygeal hamartoma in this patient (Figure 4).

## Discussion

ACD is a rare congenital malformation, with fewer than 100 cases documented in the literature. It predominantly affects pediatric populations, as most cases are identified and managed surgically during infancy or early childhood. Consequently, presentation in adolescents or adults is exceedingly uncommon. Histopathological criteria for ACD, as established by Ochiai et al., include the presence of squamous epithelium at the caudal end, transitional epithelium at the cranial end, and smooth muscle fibers within the lesion wall [6]. ACD can remain asymptomatic for many years, making early diagnosis particularly challenging. When symptoms do appear, local infection and discharge are the most commonly reported signs. In cases such as this one, where complications like infection or abscess formation occur, the clinical presentation may closely resemble more common anorectal conditions, including perianal fistulas or dermoid cysts [7].

Given the rarity and nonspecific clinical features of ACD, a high index of suspicion is necessary. Imaging plays a central role in the diagnostic process. MRI is especially valuable, not only for ruling out other presacral anomalies but also for delineating the anatomical extent of the duplication and identifying any associated fistulous tracts. It provides essential information that physical examination alone cannot reveal and is instrumental in guiding surgical planning [8]. In the present case, MRI was critical for characterizing the underlying pathology. It demonstrated a perirectal fluid collection with features suggestive of an abscess, alongside a distinct presacral mass initially raising concern for a neoplasm. Although abscess formation is an uncommon complication of ACD, its presence in this case contributed to the diagnostic complexity and further emphasized the need for comprehensive imaging and multidisciplinary evaluation. Additional diagnostic modalities, such as fistulography, can be instrumental in excluding fistulous tracts or any abnormal communication with the anal or rectal lumen. In our case, anoscopy combined with the injection of hydrogen peroxide and methylene blue was utilized to confirm the absence of such communication with the anal canal. Echoendoscopy also offers significant value in delineating the anal sphincter complex, the duplicated tract, and its dimensions [9]. Furthermore, a colonoscopy was performed to exclude inflammatory bowel disease and to rule out any fistulous connection to the rectum. ACD is often associated with other congenital anomalies, with incidence rates reported as high as 36% [2]. The most commonly reported conditions include anorectal malformations, anal duplication cysts, intestinal malrotation, sacrococcygeal teratoma, myelomeningocele, tethered cord, congenital heart defects, cleft lip/palate, and genitourinary anomalies [10]. Numerous cases of congenital perineal masses, such as hamartomas and lipomas, have been documented in association with rectal duplication. However, the association between hamartomas and anal duplication is extremely rare [11]. Interestingly, our patient exhibits a very rare association of hamartoma with anal duplication, highlighting the importance of a thorough and multidisciplinary evaluation. This approach should include input from gastroenterology, surgery, radiology, and pathology specialists to ensure a comprehensive assessment and identification of any associated abnormalities or complications.

The standard treatment for symptomatic ACD is surgical intervention. However, the management of asymptomatic cases remains a topic of debate, partly due to concerns raised by Dukes and Galvin in 1956 regarding the potential risk of malignant transformation. The primary surgical objective is the removal of the duplicated canal to prevent recurrent infections and complications such as abscess or fistula formation. Two main surgical approaches are commonly employed: mucosal stripping with closure of the remaining canal or complete excision of the duplicated anal canal [3]. In asymptomatic cases, conservative management may be an appropriate option, given the inherent risks associated with surgical intervention. In our case, however, surgical drainage of the abscess along with complete excision of the duplicated anal canal and associated mass was deemed necessary due to the presence of an abscess, persistent symptoms, and the need for histopathological confirmation of the congenital lesion.

## Conclusions

This case of a 17-year-old female patient with ACD complicated by abscess formation and an associated sacrococcygeal hamartoma adds to the limited literature on ACD. It underscores the diagnostic challenges posed by this rare condition, particularly when it mimics more common anorectal disorders. The case highlights the value of advanced imaging and endoscopic evaluation in the diagnostic process and demonstrates the necessity of a multidisciplinary approach for optimal management. Surgical resection remains the mainstay of treatment in symptomatic cases, especially with complications such as infection or other congenital complications. Early recognition and comprehensive evaluation are essential to improve outcomes and avoid misdiagnosis.
